# 4-Formyl-2-nitro­phenyl benzoate

**DOI:** 10.1107/S1600536814002694

**Published:** 2014-02-08

**Authors:** Rodolfo Moreno-Fuquen, Geraldine Hernandez, Alan R. Kennedy

**Affiliations:** a Departamento de Química, Facultad de Ciencias, Universidad del Valle, Apartado 25360, Santiago de Cali, Colombia; b WestCHEM, Department of Pure and Applied Chemistry, University of Strathclyde, 295 Cathedral Street, Glasgow G1 1XL, Scotland

## Abstract

In the title nitroaryl benzoate derivative, C_14_H_9_NO_5_, the aromatic rings form a dihedral angle of 46.37 (8)°. The central ester moiety, —C—(C=O)—O—, is essentially planar (r.m.s. deviation for all non-H atoms = 0.0283 Å) and forms a dihedral angle of 54.06 (9)° with the 4-formyl-2-nitro­phenyl ring and 7.99 (19)° with the benzoate ring. In the crystal, mol­ecules are inter­twined by weak C—H⋯O inter­actions, forming helical chains along [100].

## Related literature   

For similar esters, see: Moreno-Fuquen *et al.* (2013*a* ▶,*b* ▶, 2014 ▶). For hydrogen bonding, see: Nardelli (1995 ▶) and for hydrogen-bond motifs, see: Etter (1990 ▶).
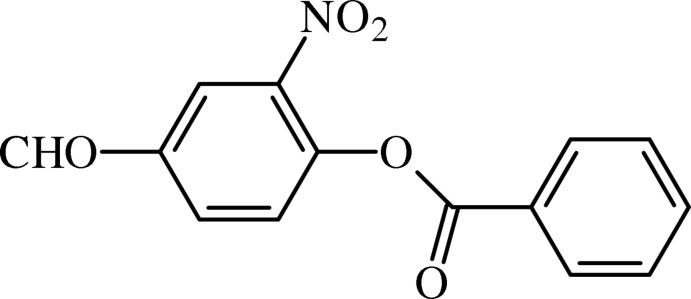


## Experimental   

### 

#### Crystal data  

C_14_H_9_NO_5_
*M* *_r_* = 271.22
Monoclinic, 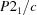

*a* = 11.3478 (11) Å
*b* = 3.7101 (5) Å
*c* = 27.723 (2) Å
β = 94.979 (9)°
*V* = 1162.8 (2) Å^3^
*Z* = 4
Cu *K*α radiation
μ = 1.02 mm^−1^
*T* = 123 K
0.21 × 0.12 × 0.02 mm

#### Data collection  

Oxford Diffraction Xcalibur E diffractometer
Absorption correction: multi-scan (*CrysAlis PRO*; Agilent, 2010 ▶) *T*_min_ = 0.813, *T*_max_ = 1.000
4231 measured reflections
2213 independent reflections
1403 reflections with *I* > 2σ(*I*)
*R*_int_ = 0.049

#### Refinement  

*R*[*F* ^2^ > 2σ(*F* ^2^)] = 0.065
*wR*(*F* ^2^) = 0.190
*S* = 0.99
2213 reflections
186 parameters
H atoms treated by a mixture of independent and constrained refinement
Δρ_max_ = 0.37 e Å^−3^
Δρ_min_ = −0.28 e Å^−3^

### 

Data collection: *CrysAlis PRO* (Agilent, 2010 ▶); cell refinement: *CrysAlis PRO*; data reduction: *CrysAlis PRO*; program(s) used to solve structure: *SHELXS97* (Sheldrick, 2008 ▶); program(s) used to refine structure: *SHELXL97* (Sheldrick, 2008 ▶); molecular graphics: *ORTEP-3 for Windows* (Farrugia, 2012 ▶) and *Mercury* (Macrae *et al.*, 2006 ▶); software used to prepare material for publication: *WinGX* (Farrugia, 2012 ▶).

## Supplementary Material

Crystal structure: contains datablock(s) I, global. DOI: 10.1107/S1600536814002694/hg5380sup1.cif

Structure factors: contains datablock(s) I. DOI: 10.1107/S1600536814002694/hg5380Isup2.hkl

Supporting information file. DOI: 10.1107/S1600536814002694/hg5380Isup3.cml

CCDC reference:

Additional supporting information:  crystallographic information; 3D view; checkCIF report

## Figures and Tables

**Table 1:** Hydrogen-bond geometry (Å, °)

*D*—H⋯*A*	*D*—H	H⋯*A*	*D*⋯*A*	*D*—H⋯*A*
C10—H10⋯O4^i^	0.95	2.50	3.343 (4)	148
C12—H12⋯O5^ii^	0.95	2.62	3.346 (4)	134

Symmetry codes: (i) 
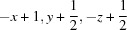
; (ii) 
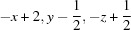
.

**Fig. 1.**
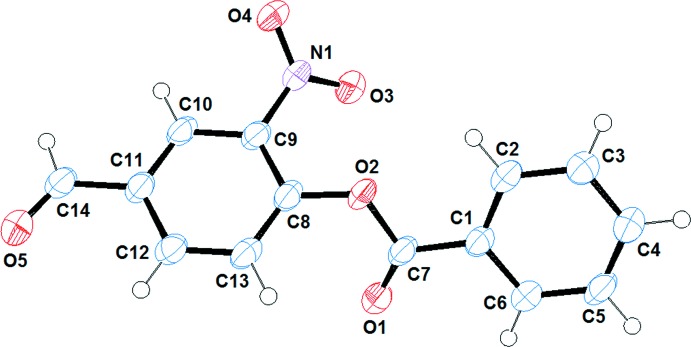
Molecular conformation and atom numbering scheme for the title compound with displacement ellipsoids drawn at the 50% probability level. H atoms are shown as spheres of arbitrary radius.

**Fig. 2.**
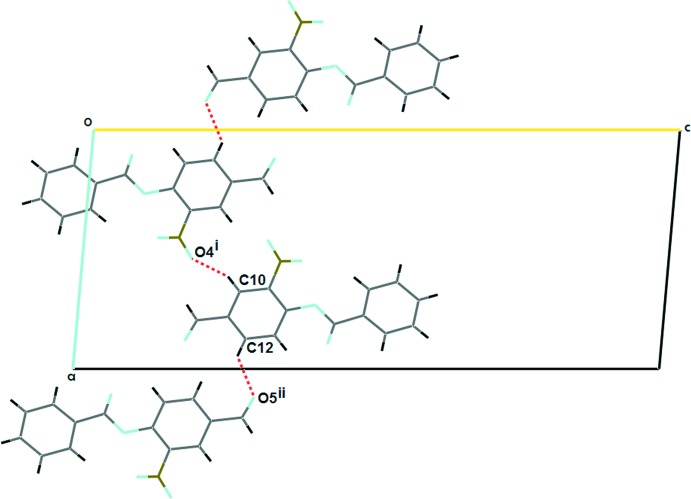
Part of the crystal structure of (I), showing the formation of helical chains which running along [100]. Symmetry code: (i) -x+1,+y+1/2,-z+1/2; (ii) -x+2,+y-1/2,-z+1/2.

## supplementary crystallographic information

### 1. Comment

The title compound 4-formyl-2-nitrophenyl benzoate (I), is part of a series of studies on the structural properties of the formyl nitro aryl benzoates developed by our research group. The molecular structure of (I) is shown in Fig. 1. Bond lengths and bond angles show marked similarity with the 4-formyl-2-nitrophenyl 4-bromo benzoate (F4BrB) (Moreno-Fuquen *et al.*, 2013*a*), 4-formyl-2-nitrophenyl 4-cloro benzoate (F4ClB) (Moreno-Fuquen *et al.*, 2013*b*) and 4-formyl-2-nitrophenyl 3-nitro-2-methyl benzoate (F3N2MB) (Moreno-Fuquen *et al.*, 2014) reported earlier jobs. The benzene rings of (I) form a dihedral angle of 46.36 (8)°. This value is quite different when compared to the systems F4BrB [62.90 (7)°], F4ClB [19.55 (9)°] and F3N2MB [4.96 (3)°]. Substituents on the rings of each system are crucial in defining the values of this angle. The central ester moiety, C1-(C7=O1)-O2-C8, is essentially planar (rms deviation for all non-H atoms = 0.0283 Å) and it forms dihedral angles of 54.06 (9)° with the formyl nitro aryl ring and 7.99 (19)° with the benzoate ring. The nitro group forms a dihedral angle with the adjacent benzene ring of 39.66 (12)°. In the crystal, the C10 and C12 atoms of the formyl nitro aryl ring at (x, y, z) act as a hydrogen-bond donors to atom O4 at (-x+1,+y+1/2,-z+1/2) and to atom O5 at (-x+2,+y-1/2,-z+1/2) forming C(5) and C(7) helical chains (Etter, 1990), along [100] (See Fig. 2). These interactions are presented in Table 1. (Nardelli, 1995).

### 2. Experimental

The reagents and solvents for the synthesis were obtained from the Aldrich-Sigma Chemical Co., and were used without additional purification. In a 25 ml round bottom flask, 4-hydroxy-3-nitrobenzaldehyde (0.201 g, 0.571 mmol) and benzoyl chloride in equimolar amounts, were dissolved in 20 mL of acetonitrile. After a short period of time, 0.03 ml of pyridine were added. Then the mixture was left to reflux in constant stirring for about two hours. A colourless solid was obtained after leaving the solvent to evaporate. m.p 384 (1)K.

### 3. Refinement

All H-atoms were positioned at geometrically idealized positions with C—H distances of 0.95 Å and U_iso_(H) = 1.2 times U_eq_ of the parent C-atoms. The H14 atom was found from difference Fourier map and its coordinates were refined freely.

### Crystal data

**Table:**

C_14_H_9_NO_5_	*F*(000) = 560
*M**_r_* = 271.22	*D*_x_ = 1.549 Mg m^−^^3^
Monoclinic, *P*2_1_/*c*	Melting point: 457(1) K
Hall symbol: -P 2ybc	Cu *K*α radiation, λ = 1.54180 Å
*a* = 11.3478 (11) Å	Cell parameters from 977 reflections
*b* = 3.7101 (5) Å	θ = 3.9–73.3°
*c* = 27.723 (2) Å	µ = 1.02 mm^−^^1^
β = 94.979 (9)°	*T* = 123 K
*V* = 1162.8 (2) Å^3^	Plate, colourless
*Z* = 4	0.21 × 0.12 × 0.02 mm

### Data collection

**Table:**

Oxford Diffraction Xcalibur E diffractometer	2213 independent reflections
Radiation source: fine-focus sealed tube	1403 reflections with *I* > 2σ(*I*)
Graphite monochromator	*R*_int_ = 0.049
ω scans	θ_max_ = 70.0°, θ_min_ = 3.9°
Absorption correction: multi-scan (*CrysAlis PRO*; Agilent, 2010)	*h* = −10→13
*T*_min_ = 0.813, *T*_max_ = 1.000	*k* = −4→4
4231 measured reflections	*l* = −33→33

### Refinement

**Table:**

Refinement on *F*^2^	Secondary atom site location: difference Fourier map
Least-squares matrix: full	Hydrogen site location: inferred from neighbouring sites
*R*[*F*^2^ > 2σ(*F*^2^)] = 0.065	H atoms treated by a mixture of independent and constrained refinement
*wR*(*F*^2^) = 0.190	*w* = 1/[σ^2^(*F*_o_^2^) + (0.0857*P*)^2^] where *P* = (*F*_o_^2^ + 2*F*_c_^2^)/3
*S* = 0.99	(Δ/σ)_max_ < 0.001
2213 reflections	Δρ_max_ = 0.37 e Å^−^^3^
186 parameters	Δρ_min_ = −0.28 e Å^−^^3^
0 restraints	Extinction correction: *SHELXL97* (Sheldrick, 2008), Fc^*^=kFc[1+0.001xFc^2^λ^3^/sin(2θ)]^-1/4^
Primary atom site location: structure-invariant direct methods	Extinction coefficient: 0.0028 (7)

### Special details

**Table:**

Geometry. All esds (except the esd in the dihedral angle between two l.s. planes) are estimated using the full covariance matrix. The cell esds are taken into account individually in the estimation of esds in distances, angles and torsion angles; correlations between esds in cell parameters are only used when they are defined by crystal symmetry. An approximate (isotropic) treatment of cell esds is used for estimating esds involving l.s. planes.
Refinement. Refinement of *F*^2^ against ALL reflections. The weighted *R*-factor *wR* and goodness of fit *S* are based on *F*^2^, conventional *R*-factors *R* are based on *F*, with *F* set to zero for negative *F*^2^. The threshold expression of *F*^2^ > σ(*F*^2^) is used only for calculating *R*-factors(gt) *etc*. and is not relevant to the choice of reflections for refinement. *R*-factors based on *F*^2^ are statistically about twice as large as those based on *F*, and *R*- factors based on ALL data will be even larger.

### Fractional atomic coordinates and isotropic or equivalent isotropic displacement parameters (Å^2^)

**Table:**

	*x*	*y*	*z*	*U*_iso_*/*U*_eq_	
O1	0.89645 (19)	0.3296 (7)	0.43051 (7)	0.0419 (7)	
O2	0.73248 (18)	0.0150 (7)	0.40350 (7)	0.0398 (6)	
O5	0.8819 (2)	−0.0584 (8)	0.18661 (8)	0.0510 (8)	
O3	0.5482 (2)	0.4580 (8)	0.37507 (8)	0.0457 (7)	
O4	0.46029 (19)	0.1854 (8)	0.31290 (7)	0.0462 (7)	
N1	0.5497 (2)	0.2761 (9)	0.33830 (9)	0.0374 (7)	
C7	0.8124 (3)	0.1534 (10)	0.43947 (10)	0.0356 (8)	
C1	0.7763 (3)	0.0601 (10)	0.48775 (11)	0.0363 (8)	
C2	0.6678 (3)	−0.1079 (9)	0.49328 (11)	0.0370 (8)	
H2	0.6164	−0.1707	0.4657	0.044*	
C3	0.6360 (3)	−0.1820 (11)	0.53964 (11)	0.0404 (8)	
H3	0.5626	−0.2956	0.5439	0.048*	
C4	0.7118 (3)	−0.0895 (10)	0.57954 (12)	0.0441 (9)	
H4	0.6897	−0.1382	0.6112	0.053*	
C5	0.8190 (3)	0.0722 (11)	0.57390 (11)	0.0436 (9)	
H5	0.8708	0.1306	0.6016	0.052*	
C6	0.8514 (3)	0.1497 (10)	0.52809 (11)	0.0393 (8)	
H6	0.9249	0.2641	0.5243	0.047*	
C8	0.7551 (3)	0.0466 (10)	0.35567 (11)	0.0353 (8)	
C9	0.6635 (3)	0.1614 (10)	0.32282 (11)	0.0345 (8)	
C10	0.6769 (3)	0.1666 (10)	0.27316 (11)	0.0364 (8)	
H10	0.6138	0.2447	0.2508	0.044*	
C11	0.7821 (3)	0.0577 (10)	0.25704 (11)	0.0369 (8)	
C12	0.8741 (3)	−0.0597 (10)	0.29011 (11)	0.0375 (8)	
H12	0.9469	−0.1342	0.2788	0.045*	
C13	0.8601 (3)	−0.0684 (10)	0.33923 (11)	0.0380 (8)	
H13	0.9224	−0.1530	0.3615	0.046*	
C14	0.7966 (3)	0.0616 (11)	0.20439 (11)	0.0399 (9)	
H14	0.731 (3)	0.180 (10)	0.1847 (11)	0.039 (9)*	

### Atomic displacement parameters (Å^2^)

**Table:**

	*U*^11^	*U*^22^	*U*^33^	*U*^12^	*U*^13^	*U*^23^
O1	0.0400 (13)	0.0533 (16)	0.0313 (12)	−0.0048 (12)	−0.0026 (10)	0.0012 (11)
O2	0.0384 (12)	0.0560 (16)	0.0233 (11)	−0.0044 (11)	−0.0075 (9)	0.0031 (11)
O5	0.0475 (15)	0.072 (2)	0.0329 (12)	0.0034 (14)	−0.0016 (11)	−0.0037 (13)
O3	0.0450 (14)	0.0584 (18)	0.0329 (12)	−0.0017 (12)	−0.0009 (10)	−0.0091 (12)
O4	0.0376 (13)	0.0667 (19)	0.0322 (12)	0.0026 (12)	−0.0085 (10)	−0.0034 (12)
N1	0.0377 (15)	0.0472 (18)	0.0259 (12)	0.0007 (13)	−0.0056 (11)	0.0023 (13)
C7	0.0355 (17)	0.043 (2)	0.0264 (15)	0.0041 (15)	−0.0074 (13)	−0.0006 (15)
C1	0.0355 (17)	0.045 (2)	0.0271 (16)	0.0055 (15)	−0.0036 (13)	0.0026 (15)
C2	0.0397 (18)	0.041 (2)	0.0283 (16)	0.0030 (15)	−0.0070 (13)	0.0004 (15)
C3	0.0380 (17)	0.047 (2)	0.0353 (17)	0.0033 (16)	0.0003 (14)	0.0018 (16)
C4	0.047 (2)	0.054 (2)	0.0310 (16)	0.0024 (18)	−0.0013 (14)	0.0038 (17)
C5	0.0449 (19)	0.055 (2)	0.0281 (16)	0.0046 (17)	−0.0122 (14)	−0.0049 (16)
C6	0.0365 (17)	0.048 (2)	0.0320 (16)	0.0003 (16)	−0.0038 (13)	0.0022 (16)
C8	0.0381 (17)	0.0407 (19)	0.0258 (15)	−0.0061 (15)	−0.0051 (13)	0.0036 (14)
C9	0.0332 (16)	0.0404 (19)	0.0285 (15)	−0.0025 (15)	−0.0051 (12)	−0.0003 (15)
C10	0.0362 (17)	0.044 (2)	0.0266 (15)	−0.0005 (16)	−0.0091 (13)	0.0055 (15)
C11	0.0383 (18)	0.044 (2)	0.0271 (15)	−0.0036 (15)	−0.0043 (13)	−0.0019 (15)
C12	0.0363 (17)	0.042 (2)	0.0333 (16)	−0.0043 (15)	−0.0043 (13)	−0.0043 (15)
C13	0.0384 (17)	0.044 (2)	0.0292 (16)	−0.0008 (15)	−0.0087 (13)	0.0034 (15)
C14	0.0382 (18)	0.050 (2)	0.0301 (17)	−0.0024 (16)	−0.0064 (14)	0.0008 (16)

### Geometric parameters (Å, º)

**Table:**

O1—C7	1.200 (4)	C4—H4	0.9500
O2—C8	1.377 (4)	C5—C6	1.383 (5)
O2—C7	1.387 (3)	C5—H5	0.9500
O5—C14	1.208 (4)	C6—H6	0.9500
O3—N1	1.224 (3)	C8—C13	1.380 (5)
O4—N1	1.231 (3)	C8—C9	1.388 (4)
N1—C9	1.459 (4)	C9—C10	1.398 (4)
C7—C1	1.475 (4)	C10—C11	1.372 (4)
C1—C6	1.387 (4)	C10—H10	0.9500
C1—C2	1.399 (5)	C11—C12	1.398 (4)
C2—C3	1.392 (4)	C11—C14	1.483 (4)
C2—H2	0.9500	C12—C13	1.385 (4)
C3—C4	1.384 (4)	C12—H12	0.9500
C3—H3	0.9500	C13—H13	0.9500
C4—C5	1.377 (5)	C14—H14	0.99 (3)
			
C8—O2—C7	119.7 (3)	C5—C6—H6	120.1
O3—N1—O4	123.9 (3)	C1—C6—H6	120.1
O3—N1—C9	118.8 (2)	O2—C8—C13	122.0 (3)
O4—N1—C9	117.3 (3)	O2—C8—C9	117.8 (3)
O1—C7—O2	122.3 (3)	C13—C8—C9	119.8 (3)
O1—C7—C1	127.2 (3)	C8—C9—C10	120.8 (3)
O2—C7—C1	110.5 (3)	C8—C9—N1	121.9 (3)
C6—C1—C2	120.2 (3)	C10—C9—N1	117.4 (3)
C6—C1—C7	118.4 (3)	C11—C10—C9	119.3 (3)
C2—C1—C7	121.4 (3)	C11—C10—H10	120.4
C3—C2—C1	119.3 (3)	C9—C10—H10	120.4
C3—C2—H2	120.3	C10—C11—C12	120.0 (3)
C1—C2—H2	120.3	C10—C11—C14	119.5 (3)
C4—C3—C2	119.7 (3)	C12—C11—C14	120.5 (3)
C4—C3—H3	120.1	C13—C12—C11	120.6 (3)
C2—C3—H3	120.1	C13—C12—H12	119.7
C5—C4—C3	120.7 (3)	C11—C12—H12	119.7
C5—C4—H4	119.6	C8—C13—C12	119.6 (3)
C3—C4—H4	119.6	C8—C13—H13	120.2
C4—C5—C6	120.2 (3)	C12—C13—H13	120.2
C4—C5—H5	119.9	O5—C14—C11	124.0 (3)
C6—C5—H5	119.9	O5—C14—H14	122.0 (19)
C5—C6—C1	119.8 (3)	C11—C14—H14	114.0 (19)
			
C8—O2—C7—O1	6.7 (5)	O2—C8—C9—N1	5.3 (5)
C8—O2—C7—C1	−174.7 (3)	C13—C8—C9—N1	178.3 (3)
O1—C7—C1—C6	−7.0 (6)	O3—N1—C9—C8	40.2 (5)
O2—C7—C1—C6	174.5 (3)	O4—N1—C9—C8	−140.5 (3)
O1—C7—C1—C2	171.6 (4)	O3—N1—C9—C10	−140.6 (3)
O2—C7—C1—C2	−6.9 (5)	O4—N1—C9—C10	38.6 (5)
C6—C1—C2—C3	0.3 (5)	C8—C9—C10—C11	−0.2 (5)
C7—C1—C2—C3	−178.2 (3)	N1—C9—C10—C11	−179.4 (3)
C1—C2—C3—C4	−0.1 (5)	C9—C10—C11—C12	0.5 (5)
C2—C3—C4—C5	−0.6 (6)	C9—C10—C11—C14	179.8 (3)
C3—C4—C5—C6	1.1 (6)	C10—C11—C12—C13	0.2 (5)
C4—C5—C6—C1	−0.8 (6)	C14—C11—C12—C13	−179.1 (3)
C2—C1—C6—C5	0.1 (6)	O2—C8—C13—C12	174.3 (3)
C7—C1—C6—C5	178.7 (3)	C9—C8—C13—C12	1.6 (5)
C7—O2—C8—C13	53.3 (5)	C11—C12—C13—C8	−1.3 (5)
C7—O2—C8—C9	−133.8 (3)	C10—C11—C14—O5	−173.3 (4)
O2—C8—C9—C10	−173.9 (3)	C12—C11—C14—O5	5.9 (6)
C13—C8—C9—C10	−0.8 (5)		

### Hydrogen-bond geometry (Å, º)

**Table:**

*D*—H···*A*	*D*—H	H···*A*	*D*···*A*	*D*—H···*A*
C10—H10···O4^i^	0.95	2.50	3.343 (4)	148
C12—H12···O5^ii^	0.95	2.62	3.346 (4)	134

Symmetry codes: (i) −*x*+1, *y*+1/2, −*z*+1/2; (ii) −*x*+2, *y*−1/2, −*z*+1/2.
